# Repeated Evacuation of the Aqueous Humour in Diseases of the Eye

**Published:** 1866

**Authors:** Casimir Sperino


### Repeated Evacuation of the Aqueous Humour in Diseases of the Eye.

BY CASIMIR SPERINO.

He says: I use myself a small two-edged knife, very slightly curved on the flat, and having a slight ridge on each side, so as to render its faces convex from edge to edge, and to facilitate penetration. The width of the blade is about three millimetres. This knife is carried into the anterior chamber, with the concave face of the blade to the front, at any selected point of the circumference of the cornea, or even encroaching a little on the margin of the sclerotic, where it overlaps the cornea. The blade being withdrawn, the discharge of the aqueous humor is effected by means of a blunt probe, of metal or whalebone, which is carried more or less deeply into the anterior chamber, and then pressed lightly backwards.

The place of election for the puncture is nearly always the periphery of the cornea, more especially when it is intended to repeat the evacuations by the same opening. In ladies, however, I have made the opening in the sclerotic border, so as to leave no trace of a cicatrix. The point of the periphery is a matter of indifference, unless the chamber contain morbid products, blood, or pus, which may escape more readily at one part than at another. The aqueous humor escapes with equal readiness from all.

When the puncture is made through the sclerotic margin, it is usual to witness the escape of a few drops of blood, proceeding either from Shlemm's canal, or from the vessels entering it, and more abundant when the radiating peri-corneal vascular circle is injected, or when there is congestion of the anterior portion of the choroid. The conjunctiva itself may also furnish a little blood; and the bleeding from both these sources may recur, for one or two days, at each introduction of the probe. Such bleeding has never been attended by any mischance, and has often appeared to render the improvement more rapid.

The sole objection to sclerotic puncture rests upon the tendency of the opening to be obscured by a slight swelling of the surrounding conjunctiva; so that successive introductions of the probe are rendered difficult, and, if the eye be very sensitive, painful. It is important that the opening should not be too small, that it should be in a place easy of discovery, and that this place should be accurately remembered by the operator. The puncture, however, is in no way dangerous, and may be repeated as often as may be necessary. In my own practice, the place chosen is on the temporal side, and in the horizontal meridian of the cornea, either at the corneo-scleral junction, or about one millimetre farther back. The course of the knife is in a line parallel to the plane of the iris, which is thus never in danger of being wounded. By selecting always the same point of puncture, it is found easily and almost instinctively, for the purpose of successive evacuations by the probe.

If the opening made be too large, it will sometimes occasion a prolapse of the iris after the escape of the aqueous humor. This accident must be treated in the ordinary way. I have sometimes profited by it to excise a piece of the iris, or to displace the pupil. Otherwise, gentle friction upon the closed lid, the pressure of a Daviel's spoon upon the prolapse, or the exposure of the eye to light, will usually effect reduction.

The only serious mischance which, in unskilful hands, can result from the puncture, is injury to the crystalline lens. By ordinary care such an accident may be rendered impossible. The knife should be held parallel to the plane of the iris, steadily introduced, and rapidly withdrawn in the same direction. The aqueous humour does not escape during this step of the operation, and neither the lens nor the iris can be wounded. Wounds of the iris, however, although well to be avoided, are seldom productive of any inconvenience.

As the anterior chamber is emptied, the iris gradually advances until it comes into complete contact with the cornea, and the tension of the globe diminishes in a degree proportionate to the quantity of the aqueous humour. If the state of the anterior chamber be normal, the pupil dilates a little, yielding to the forward pressure of the lens. After a few minutes the chamber is refilled as before; although the rapidity with which the aqueous humor is restored varies much in the various diseases of the eye. In order to empty the chamber again, the wound is re-opened by a little probe, the simplest introduction of which is often sufficient. Sometimes it is necessary to use a little backward pressure, since the iris, closely applied to the cornea at the puncture, may impede the escape of the fluid. Such pressure is the only part of the procedure that may be a little painful, by its influence upon the ciliary circle and plexus of nerves. The sensitiveness of these parts is a reason for not losing sight of the exact place of puncture, and for not making painful exploration with the probe for its discovery. An increase of sensitiveness in the eye is never to be considered a contra-indication for paracentesis; since such sensitiveness and the accompanying tension and pain, are nearly always removed by successive evacuations.

The forward pressure of the iris and lens is the immediate cause of the escape of the aqueoue humor. When there is posterior synechia, this pressure is not exercised, and the fluid no longer escapes spontaneously. It is then necessary to introduce the probe repeatedly, and to introduce a stronger pressure, in order to cause the escape of the humour. During the first days of the treatment, it is often impossible to empty the anterior chamber completely.

When the chamber contains concrete pus, or lymph, it is sometimes necessary to carry the probe across the morbid product, in order to cause the escape of the humour; and, under similar circumstances, it may be needful to introduce the probe several times, especially if care has not been taken to make an opening sufficiently large. In such cases the blunt whalebone probe should be preferred, since it is less liable to bruise, or injure the iris. * * *

The evacuations of the aqueous humour by the same apperature may be effected by two methods—either repeatedly in a single *seance*, followed by an interval of some days, or singly, at shorter intervals, and for a considerable time.

By the first method the anterior chamber is emptied, at the same *seance* and by the same opening, twice, thrice, four or even more times, at intervals of some minutes, according to the quantity of the fluid and the rapidity of its reproduction. By the second method, the probe is introduced and the chamber emptied in the morning, and again in the evening, or several times a day, and this for many days, or even weeks. Sometimes it may be necessary to combine these methods, and sometimes it will suffice to empty the chamber once every day.

Commonly, after thirty or forty hours, the corneal opening is found closed, and it is necessary to use a little pressure with the probe, in order to break down the adhesions. Excepting for the trifling pain, the wound may be re-opened after an interval of eight or ten days, without the least inconvenience.—*Ophthalmic Review, London.*

